# Engineering Advanced Drug Delivery Systems for Dry Eye: A Review

**DOI:** 10.3390/bioengineering10010053

**Published:** 2022-12-31

**Authors:** Tian-Zuo Wang, Xin-Xin Liu, Si-Yu Wang, Yan Liu, Xin-Yang Pan, Jing-Jie Wang, Kai-Hui Nan

**Affiliations:** 1 State Key Laboratory of Ophthalmology, Optometry and Vision Science, School of Ophthalmology & Optometry, Wenzhou Medical University, Wenzhou 325027, China; 2 National Clinical Research Center for Ocular Diseases, Wenzhou 325027, China; 3 National Engineering Research Center of Ophthalmology and Optometry, School of Biomedical Engineering, Wenzhou Medical University, Wenzhou 325027, China

**Keywords:** dry eye disease, drug delivery, new dosage forms, treatment

## Abstract

Dry eye disease (DED) is a widespread and frequently reported multifactorial ocular disease that not only causes ocular discomfort but also damages the cornea and conjunctiva. At present, topical administration is the most common treatment modality for DED. Due to the existence of multiple biological barriers, instilled drugs generally exhibit short action times and poor penetration on the ocular surface. To resolve these issues, several advanced drug delivery systems have been proposed. This review discusses new dosage forms of drugs for the treatment of DED in terms of their characteristics and advantages. Innovative formulations that are currently available in the market and under clinical investigation are elaborated. Meanwhile, their deficiencies are discussed. It is envisioned that the flourishing of advanced drug delivery systems will lead to improved management of DED in the near future.

## 1. Introduction

Dry eye disease (DED) is resulted from decreased tear production, excessive evaporation of tears, or both, ultimately leading to inflammation of the ocular surface [1]. It is manifested by diverse and complex pathological processes. Presently, the association of these processes is not fully established. In 1995, the American Academy of Ophthalmology (AAO) classified DED into two subtypes, namely evaporative dry eye and aqueous-deficient dry eye. However, most patients with DED present symptoms of both subtypes [2]. Epithelial lesions of the ocular surface, inflammation, and neurosensory abnormalities caused by DED can lead to the manifestation of diverse clinical symptoms, such as redness, pain, blurred vision, and sleep disorders. These discomforts not only reduce the patients’ productivity but also seriously affect their quality of life [3,4]. Epidemiological studies reveal that the prevalence of DED is approximately 7% in Europe and America, 12.5–21.6% in Japan and Republic of Korea, and 21–30% in China. In some areas of Russia, the prevalence has been reported to be 40–55% [5,6,7,8,9]. Aging, female gender, prior eye surgery, and widespread use of video terminals have been reported as high-risk factors contributing to the development of DED [10]. China has a huge and aging population, where the prevention and treatment of DED are discouraging. With the significant progress in epidemiological studies of DED in recent years, it is recognized that DED has become an important public health concern [11,12].

**Figure 1:**
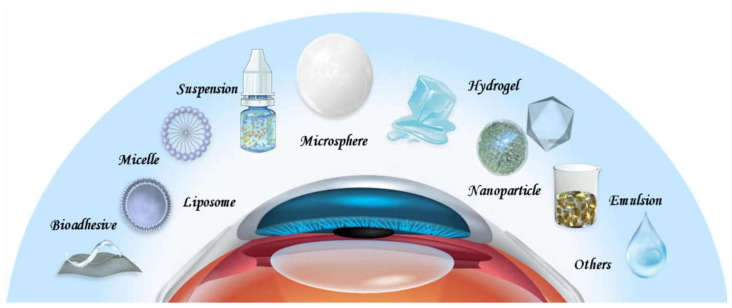
Drug delivery systems for DED.

Presently, various treatment modalities, such as warm compresses, meibomian gland expression, intranasal tear neurostimulation, contact lenses, and topical medications have been prescribed for DED, among which the topical application of medications represents the most important one [13,14,15,16]. However, multiple biological barriers on the ocular surface, such as the tear film barrier and the corneal and conjunctival barrier, result in rapid drug clearance and low bioavailability. Specifically, the presence of the tear film leads to rapid drug loss and the dense epithelium of the conjunctiva hinders drug entry [17]. Cornea is in fact an amphiphilic tissue, the epithelium layer of which is hydrophobic, so it is difficult for hydrophilic drugs to stay or diffuse. However, if the drug has high lipophilicity, it is difficult for it to penetrate the hydrophilic stromal layer. As a result, drugs must have balanced hydrophilicity and lipophilicity to pass through the entire cornea [18]. These determine that topically applied drugs generally demonstrate extremely low bioavailability. To improve the bioavailability of drugs in the eye, numerous drug delivery systems have been developed (Figure 1) [19,20,21]. For example, nanoparticles are employed to improve the corneal penetration of drugs, hydrogels are employed to extend the retention time of drugs on the ocular surface, and microspheres are employed to achieve sustained release. While eye drops remain the most common delivery vehicles for DED drugs, drug-loaded implants, drug sprays, and microneedles are increasingly being explored. DED is characterized by a vicious cycle triggered by the multifactorial disruption of the ocular surface microenvironment, which leads to inflammation and decreased tear film stability [22]. To ameliorate this chronic condition, long-term treatments are inevitable. However, the long-term use of medications can be associated with serious side effects. The DEWS II report suggests that short-term and long-term treatments should be combined flexibly for different patient conditions to maximize the therapeutic benefits [23]. For patients with mild DED, the use of artificial tears alone can achieve satisfactory therapeutic effects. In contrast, for patients with moderate-to-severe DED, single or multiple drugs are often warranted. As a result, there is an attempt to improve the delivery of drugs with rationally designed drug delivery systems according to the properties of intended drugs and the disease conditions for better outcomes. Some of these delivery systems have led to innovative therapies which are under clinical investigation or even enter the clinic (Table 1) [21,24,25,26,27]. In this contribution, we will elaborate advanced drug delivery systems which are developed for DED. With recent advances in this field, it is expected that improved management of DED can be achieved in the near future.

## 2. Drug Delivery Systems for DED

### 2.1. Suspensions

Suspension refers to a liquid formulation formed by dispersing insoluble drug particles in a liquid medium. Common methods for the preparation of suspension include direct dispersion, precipitation, and controlled flocculation. Drug particles in the suspension settle slowly at a rate that does not interfere with correct dosing. They do not agglomerate and can be dispersed evenly through shaking, even after long-term storage. After topical application, drug particles in the suspension can be retained in the cul-de-sac, which leads to prolonged ocular action duration [28]. The hydrophobic drugs currently used for the treatment of DED are often administered in this manner.

Alrex^®^ (Bausch & Lomb, Clearwater, FL, USA) is an FDA-approved loteprednol etabonate suspension primarily used as an anti-inflammatory agent. Clinical trials have confirmed that DED can be treated effectively by monotherapy with this drug or combination therapy containing artificial tears [29,30]. Furthermore, suspensions of the immunosuppressant cyclosporine and the mucin-stimulating drug rebamipide have also been approved for treating DED [22,31]. In a randomized multicenter phase III study, 2% rebamipide suspension and 0.1% sodium hyaluronate were randomly instilled in 188 patients with DED, which demonstrated that the 2% rebamipide suspension was more effective in relieving foreign body sensation as well as eye pain [32].

**Figure 2:**
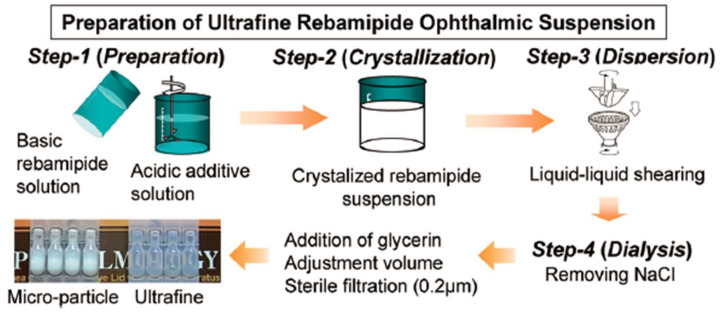
Schematic illustration for the preparation of rebamipide ultrafine suspension. Reprinted with permission from [33]. Copyright 2017 The Pharmaceutical Society of Japan.

Although rebamipide suspension is advantageous for the treatment of DED, it is a milky liquid and blurs the patient’s vision temporarily, which reduces visual quality. To overcome this shortcoming, Matsuda et al. [33] formulated rebamipide particles into an ultrafine state (approximately 640 nm in size) to obtain a highly transparent (light transmittance: 59%) suspension (Figure 2). The duration of blurred vision was thus reduced. Moreover, an in vivo pharmacokinetics study revealed that the concentrations of rebamipide in cornea and conjunctiva were higher than those of conventional suspension, which indicated accelerated absorption rates and improved bioavailability. The particle size and transparency of this suspension remain unchanged for 3 years when stored at 25 °C, which demonstrated its excellent physicochemical stability. Augmenting the mucus-penetrating abilities of drugs represents another strategy to obtain improved outcomes. In this regard, Eysuvis^®^ (Kala Pharmaceuticals, Arlington, MA, USA) is an original loteprednol nanosuspension developed by Kala using the AMPPLIFY mucus-penetrating particle drug delivery technology. This technology permits loteprednol to reach the ocular surface without being degraded in the tear film. A single application of Eysuvis^®^ can increase the concentrations of loteprednol in the aqueous humor, cornea, and conjunctiva by up to three times compared with the commercial product Lotemax^®^ (0.38% loteprednol etabonate eye gel, Bausch & Lomb, Clearwater, FL, USA). Eysuvis^®^ showed high efficacy in DED treatment [34,35]. Moreover, a multicenter randomized clinical trial demonstrated it was safe and well-tolerated for short-term use (2–4 weeks) [36]. However, the long-term in vivo safety of it remains to be determined.

### 2.2. Emulsions

Emulsion refers to a two-phase liquid in which the two phases are immiscible with each other. It generally consists of an aqueous phase (denoted by W), an oil phase (denoted by O), and an emulsifier. One phase is dispersed in the other in the form of small droplets, leading to a heterogeneous dispersion. The oil-in-water (O/W) emulsion serves as a good delivery vehicle for hydrophobic ophthalmic drugs, because the oil phase of it can be harnessed to dissolve poorly water-soluble molecules. As a result, improved ocular bioavailability is obtained [37]. Restasis^®^ (Allergan, Waco, TX, USA), an O/W anionic nanoemulsion containing cyclosporine, is the first commercially available ophthalmic emulsion for DED. It is obtained by dissolving cyclosporine in castor oil using polysorbate as the emulsifier. The drug droplets of Restasis^®^ spread easily on the ocular surface after instillation, thus allowing fast drug absorption and onset of action [38]. Emulsions also increase the solubility of hydrophobic drugs and offer ease of scale production in industrial settings [39].

Ikervis^®^ is an O/W cationic nanoemulsion of cyclosporine developed by Santen Pharmaceutical (Osaka, Japan). The drug concentration of Ikervis^®^ is the highest in the clinic. It is used in patients with severe DED that cannot be ameliorated by artificial tears. Because the emulsion droplets in Ikervis^®^ are smaller than those in Restasis^®^, the penetration of cyclosporine into the cornea is enhanced. Moreover, the unique cationic Ikervis^®^ can interact electrostatically with the negatively charged ocular surface, which prolongs residence time of the drug. The dosing frequency of Ikervis^®^ is one time per day. This has been associated with improved patient compliance and therapeutic efficiency. A good safety profile of Ikervis^®^ had also been demonstrated in a 12-month multicenter double-blind clinical trial [40]. Cationic emulsions are prone to cause local side effects, such as mild pain at the instillation site [41,42]. To improve eye comfort and bioavailability of the drug, Bang et al. developed self-emulsifying nanodrug delivery systems, namely, Cyporin-N (SNEDDS-N; Taejoon Pharma, Seoul, Republic of Korea) and T-sporin (SNEDDS-T; Taejoon Pharma, Seoul, Republic of Korea). SNEDDS is an anhydrous homogeneous mixture of oils, drugs, surfactants, and cosurfactants. Compared with the high turbidity and unstable pH of Restasis^®^, SNEDDS exhibits more uniform particle sizes, better light transmission, and more stable pH. The ability of SNEDDS to restore tear film stability is also superior to that of Restasis^®^ [43]. Currently, SNEDDS has been developed in a variety of forms and diseases, such as solid SNEDDS, controlled-release SNEDDS, mucus-permeable SNEDDS, and targeted SNEDDS [44,45,46]. It is hoped that these innovative formulations will lead to a breakthrough in the management of DED. Apart from drug-loaded emulsions, drug-free emulsions have also been developed for DED. For instance, Cationorm^®^ from Santen Pharmaceutical is a drug-free O/W cationic nanoemulsion. The benzylcetyldimethylammonium chloride presented in Cationorm^®^ is a cationic surfactant that possesses intrinsic antimicrobial activity, which makes Cationorm^®^ a preservative-free dosage form. This design enhances the safety of Cationorm^®^. Pharmacodynamic studies revealed that Cationorm^®^ not only exhibited moisturizing and lubricating properties but also stabilized the tear film by its oily components, thus making it a safe and effective tear supplement [47,48].

### 2.3. Liposomes

Liposomes are tiny vesicles of 10–1000 nm which are consisted of natural or synthetic phospholipid bilayers. They can be classified as small unilamellar vesicles, large unilamellar vesicles, giant unilamellar vesicles, oligolamellar vesicles, multilamellar large vesicles, and multivesicular vesicles [49]. Film hydration, reverse phase evaporation, solvent injection, detergent removal, and the heating method are conventionally used for the preparation of liposomes. Meanwhile, new technologies such as microfluidic methods and the supercritical fluidic method have gained considerable attention [50]. Liposomes improve the delivery of ophthalmic drugs by encapsulating hydrophobic ones in phospholipid bilayers or encapsulating hydrophilic ones in the aqueous core. With similar structure and composition as the cell membrane, liposomes are generally biodegradable and well-tolerated [51,52,53].

**Figure 3:**
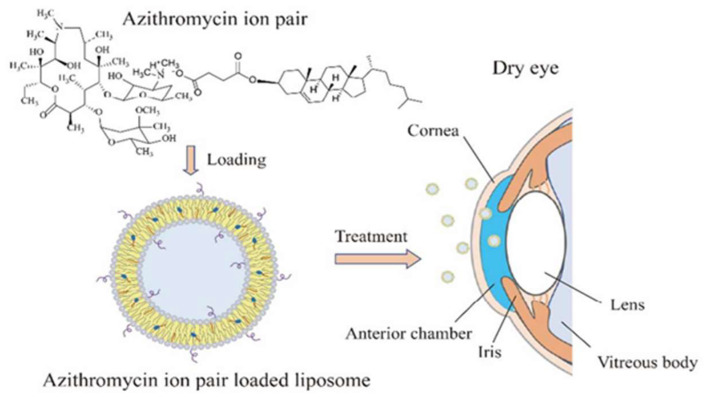
Schematic illustration for the preparation of adriamycin liposomes. Reprinted with permission from [57]. Copyright 2018 American Chemical Society.

The Tears Again^®^ liposome spray developed by Optima Pharmazeutische (Hallbergmoos, Germany) is a new generation of liposome supplements capable of repairing all three layers of the tear film. The phospholipids in the ingredients repair the lipid layer of the tear film, the isotonic solution replenishes the aqueous layer, and the sodium hyaluronate serves as an alternative to the mucus layer. Upon application, the active ingredients are absorbed transdermally. Meanwhile, the preservatives are blocked outside the skin and do not enter the tear film, thereby preventing damage to the eyes. In a double-blind clinical trial, the Tears Again^®^ liposome spray was found to reduce discomfort and stabilize the tear film more efficiently than saline spray and 0.1% sodium hyaluronate [54,55]. In another example, Chen et al. prepared tacrolimus-loaded cationic liposomes using the film hydration method. The resultant formulation prolongs the retention time of FK506 on the ocular surface, increases its concentration in the cornea, and exerts a good therapeutic effect owing to its anti-inflammatory property and ability to promote epithelial cell healing [56]. Ren T et al. formulated adriamycin ion pair loaded liposomes to improve the therapeutic effects of the drug against DED (Figure 3). First, an adriamycin–cholesterol hemisuccinate ion pair was prepared to improve the drug loading. Second, liposomes were prepared by a film hydration method. Finally, the liposomes were sonicated to obtain uniform particle size with high drug loading efficiency [57]. Recently, the macromolecular protein lactoferrin and the antioxidant astaxanthin have also been encapsulated in liposomes, which showed favorable therapeutic effects against DED as proved by in vivo pharmacodynamics studies [58,59,60].

### 2.4. Nanoparticles

Nanoparticles for drug encapsulation are nanosized delivery vehicles with particle sizes in the range of 1–1000 nm. The majority of them in ophthalmic applications are made from natural or synthetic polymers (gelatin, silk fibroin, chitosan, PLGA, PLA, PCL, etc.). Various methods, such as nanoprecipitation, self-assembly, ionic gelation, and desolvation, have been developed for their preparation. Superior therapeutic effects against ocular diseases are obtained via different mechanisms including increasing the dissolution/solubility of poorly water-soluble drugs, affording sustained release, enhancing ocular retention/penetration, or transporting drugs to targeted tissues/cells [35].

The residence time of eye drops is short due to blink and nasolacrimal duct drainage, which lead to low drug bioavailability. To overcome this drawback, Nagai et al. fabricated a rebamipide-based solid nanoparticle formulation (REB-NPs) via grinding. The thus-obtained rebamipide nanoparticles were elliptical, with particle sizes in the range of 40–200 nm. After being applied to the eyelid, rebamipide can be delivered to the tear film through meibomian glands. The rebamipide nanoparticles prolonged the release duration of rebamipide compared with the conventional rebamipide suspension. Thus, increased mucin levels and tear film restoration were obtained [61]. As the vicious cycle of DED is generally accompanied with a series of changes in the ocular surface microenvironment, nanoparticles with dual or multiple functions are likely to produce synergistic effects and lead to improved therapeutic outcomes. Li YJ et al. developed an AF/Au@Poly-CH nanoparticle formulation with anti-inflammatory and antioxidant functions. AF/Au@Poly-CH was obtained via a one-step self-assembly process. In vivo safety and efficacy studies in rabbit eyes revealed that the resultant nanoformulation was well-tolerated and demonstrated high therapeutic efficacy [62].

Drug-loaded nanoparticles have also been formulated into dosage forms other than eye drops. For example, Ryu et al. developed nanoparticles incorporated tablets, by embedding PLGA nanoparticles containing dexamethasone in an alginate matrix. The table was applied to the ocular surface using a preocular applicator. It was found that the nanoparticles remained on the ocular surface for up to 2 h. This mode of administration not only improves the bioavailability of drugs but also enables their sterile delivery [63,64]. Nanocapsules are nanoparticles with hollow cores, which are mainly used to deliver labile drugs or engineered for targeted delivery. Although several nanocapsule-based delivery systems exist for anti-inflammatory drugs, few reports are available with respect to their application for the treatment of DED [65,66]. Zhang et al. prepared cyclosporine lipid nanocapsule eye drops with the phase-inversion method, which increased bioavailabilities of cyclosporine in the conjunctiva and cornea. In line with the pharmacokinetic study, superior therapeutic effects over conventional cyclosporine emulsion were observed in pharmacodynamic studies [67].

### 2.5. Microspheres

**Figure 4:**
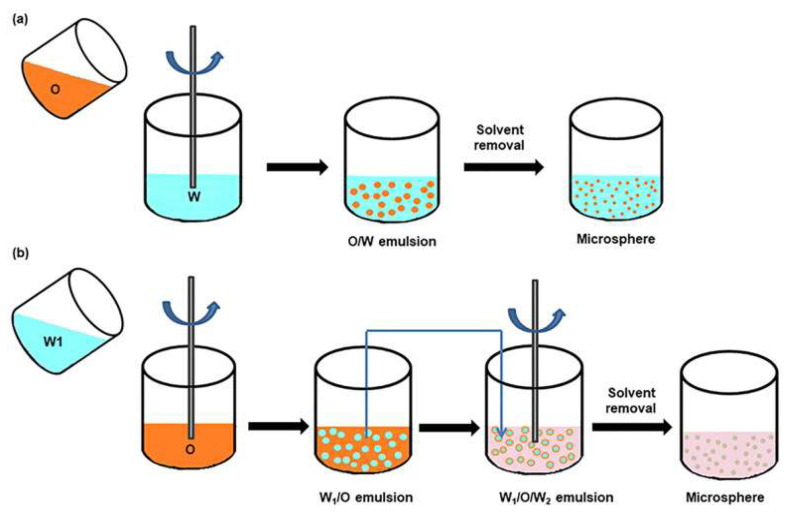
Schematic illustration of the (**a**) single emulsion method and (**b**) multiple emulsion method for microsphere preparation. Reprinted from [68] under Creative Commons Attribution License.

Microspheres are micro-sized polymeric or inorganic particles with a spherical geometry, which deliver therapeutic cargoes by adsorbing them on the particle surface or encapsulating them within the particle matrix. They have been fabricated by various techniques, such as emulsion–solvent evaporation, spray drying, phase separation, and ionic gelation, with emulsion–solvent evaporation being the most common one (Figure 4 [68]). Industrial production of microspheres is simple and cost-effective. Moreover, long-term sustained release and modular delivery of multiple drugs for combination therapy can be achieved. For moderate and severe DED, the efficiency of conventional dosage forms of anti-inflammatory drugs is insufficient. Consequently, long-term, frequent dosing of high-concentration dosage forms is required, which destroys immune homeostasis and results in numerous side effects [69]. To solve these problems, Ratay et al. formulated degradable polymer microspheres (TRI) delivering T_reg_-inducing drugs to stimulate the endogenous production of T_reg_ cells [70]. The microsphere formulation has been shown to be effective in preventing tear loss and maintaining goblet cell density. In addition, it reduces corneal fluorescein staining. However, this formulation is composed of three kinds of microspheres with different drugs, which makes the production and storage of it not cost-effective for practical application [71]. To this end, sulfanilide hydroxamic acid-loaded PLGA microspheres with a similar T_reg_-inducing function have been prepared [72]. The resultant formulation offers sustained release, inhibits inflammation, and promotes the restoration of immune homeostasis by increasing endogenous T_reg_ cells. As T_reg_ cells are intrinsic components of the immune system, it is expected that this therapeutic strategy will not disturb the immune homeostasis of the patients as conventional anti-inflammatory therapies. Although the microspheres represent a promising delivery system for DED drugs, the drawbacks of low encapsulation efficiency, poor stability, and burst release of drugs remain to be resolved.

### 2.6. Micelles

**Figure 5:**
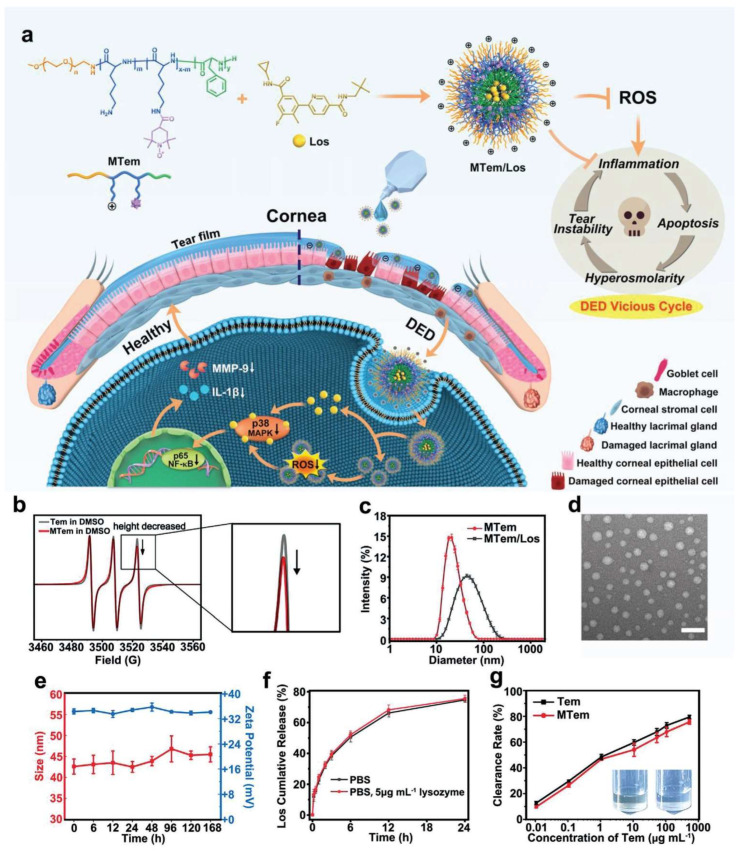
Schematic illustration for the preparation (**a**) and characterization (**b**–**g**) of anti-inflammatory and antioxidant micelles. Reprinted from [80] under Creative Commons Attribution License.

Micelles represent a class of drug delivery vehicles formulated with surfactants or amphiphilic polymers, the structure of which is characterized by a hydrophilic shell and a hydrophobic core. Therefore, hydrophobic drugs can be encapsulated in the cores for improved therapeutic effects. In regard to ocular drug delivery, the adhesive properties of polymeric micelles can prolong the residence time of drugs on the ocular surface, whereas their small sizes enhance the tissue penetration performance of drugs [73]. Notably, the aqueous solution of micelles is generally transparent, which will not interfere with the patient’s vision after application. Common techniques for micelle preparation include thin-film hydration, dialysis, lyophilization, and emulsion [74]. Micelles with uniform particle sizes can be obtained when the concentrations of surfactants or amphiphilic polymers reach their critical micelle concentrations. The high loading efficiency and ease of surface modification make micelles particularly appealing for ocular drug delivery [75,76]. Cequa^®^ is a micellar eye drop formulation of cyclosporine developed by Sun Pharmaceutical (Mumbai, India). By encapsulating the hydrophobic drug in micelles of 12–20 nm, the concentration of dissolved cyclosporine in the eye drop is greatly increased. The lyophilized powder of Cequa^®^ is stable for at least 3 months. Moreover, it was reported that the micelle formulation showed a 4.5-fold increase in ocular retention compared to the 0.05% cyclosporine emulsion [77]. Due to improved physicochemical stability and tissue penetration ability of cyclosporine, increased bioavailability was obtained [78]. It should be noted that the safety of Cequa^®^ was only evaluated in a 12-week study, which was much shorter than the conventional 12-month DED treatment duration [79]. Therefore, the long-term safety of Cequa^®^ for DED warrants further clinical evaluation. In addition to the above-mentioned marketed micelle-based formulation, micelles with dual therapeutic effects have also been developed. As shown in Figure 5, Li S et al. fabricated losmapimod-loaded polymeric micelles with cationic amphiphilic antioxidant peptides by the dialysis method. The thus-obtained formulation possessed anti-inflammatory and antioxidant activities. Ex vivo and in vivo studies revealed that it was safe for ocular application and demonstrated impressive therapeutic effects [80]. Therefore, designing multifunctional micellar drug delivery systems targeting several aspects of DED pathogenesis represents a promising direction to develop innovative therapies for DED.

### 2.7. Bioadhesive Polymers

Bioadhesion, in the pharmaceutical context, refers to a scenario in which certain high molecular polymers adhere on the mucous membranes of the mouth, nose, eye, vagina, or digestive tract [81]. The moist atmosphere of the mucous membranes can result in the swelling of bioadhesive polymers, which interpenetrate with the mucus subsequently and lead to extended tissue adhesion. This phenomenon has been widely used to prolong the in vivo residence time of drugs [82]. Most of currently used bioadhesive polymers are biodegradable and biocompatible. Furthermore, many of them have lubricant and hygroscopic properties, which aid in ameliorating the dryness of DED [83]. Bioadhesive polymers are not only used alone in monotherapies of DED, but also used as adjuvants in combination therapies. In addition, they have been employed as matrix materials to formulate other drug delivery vehicles for DED, such as nanoparticles, hydrogels, and liposomes [84,85]. Table 2 presents common bioadhesive polymers used for the management of DED. Apart from conventional bioadhesive polymers, there are also attempts to develop new bioadhesive compositions, for example, GlicoPro^®^ is an eye drop formulation based on snail mucus extracts, which demonstrates superior bioadhesive properties over sodium hyaluronate. It not only promotes corneal wound healing, but also exerts anti-inflammatory and analgesic effects [86]. Despite the promising observations, further safety and efficacy evaluations with respect to GlicoPro^®^ are warranted. In another attempt, Liu et al. designed cationized hyaluronic acid-coated spanlastics (CHASV) for ocular delivery of cyclosporine. CHASV demonstrates favorable wettability and bioadhesive properties, as well as offers sustained release of cyclosporine to the ocular surface, which is associated with improved therapeutic outcomes against DED. This novel formulation serves as an appealing alternative to commercial cyclosporine emulsions for the treatment of DED in the future [87].

### 2.8. Hydrogels

Hydrogels are a class of water-swollen network structures with high water content and mechanical properties mimicking the extracellular matrix and soft tissues. Therefore, they have received considerable attention in drug delivery and tissue engineering. Hydrogels are traditionally formulated by physical or covalent cross-linking of hydrophilic polymers [88]. Nowadays, there are also increasing instances of hydrogels obtained from the self-assembly of small molecules. As for DED, in situ gels and ocular implants are among the most common subtypes of hydrogels for drug delivery. The viscous properties of in situ gels can be harnessed to prolong the ocular residence time of drugs. On the other hand, ocular implants, such as contact lenses, serve as drug reservoirs to provide sustained release.

#### 2.8.1. In Situ Gels

**Figure 6:**
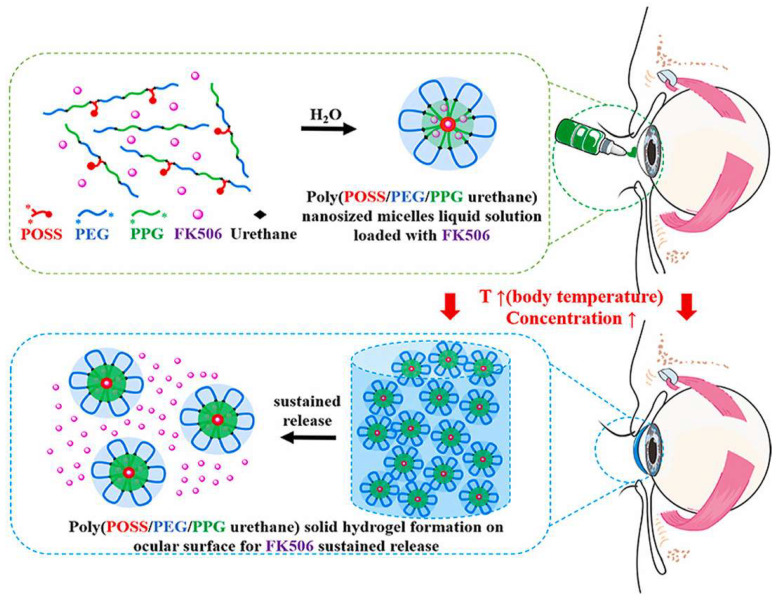
Schematic illustration for the preparation of FK506-encapsulated temperature-sensitive hydrogel. Reprinted from [91] under the CC BY-NC-ND license.

In situ gels are a class of hydrogels that undergo sol–gel transition after in vivo application. They are also known as smart hydrogels as the phase transition can be triggered by various physiological signals, such as temperature, pH, or ions in the tear fluid [89]. The sol–gel transition leads to a bioadhesive network that fixes the drugs on the ocular surface, which prolongs ocular retention and facilitates sustained release. A recent study revealed that levocarnitine delivered by in situ gels showed superior therapeutic effects against DED [90]. Han Y et al. encapsulated FK506 in a thermos-responsive in situ gel formulated with POSS, PEG, and PPG (Figure 6). The resultant formulation was biocompatible. Moreover, prolonged ocular retention and enhanced therapeutic efficiency were demonstrated in a mouse DED model [91]. Eldesouky et al. developed a thermosensitive in situ gel containing lipid nanocapsules loaded with cyclosporine. This dosage form can not only prolong the ocular retention of cyclosporine, but also enhance its tissue penetration ability. A further pharmacodynamic study revealed it outperformed the commercial cyclosporine nanoemulsion in restoring tear production of DED rabbits [92]. The work of Eldesouky et al. provides a promising alternative for DED. However, the use of surfactants and organic solvents during hydrogel fabrication may cause irritation. Therefore, the biocompatibility of this dosage form should be evaluated thoroughly for its successful clinical application.

#### 2.8.2. Hydrogel Implants

**Figure 7:**
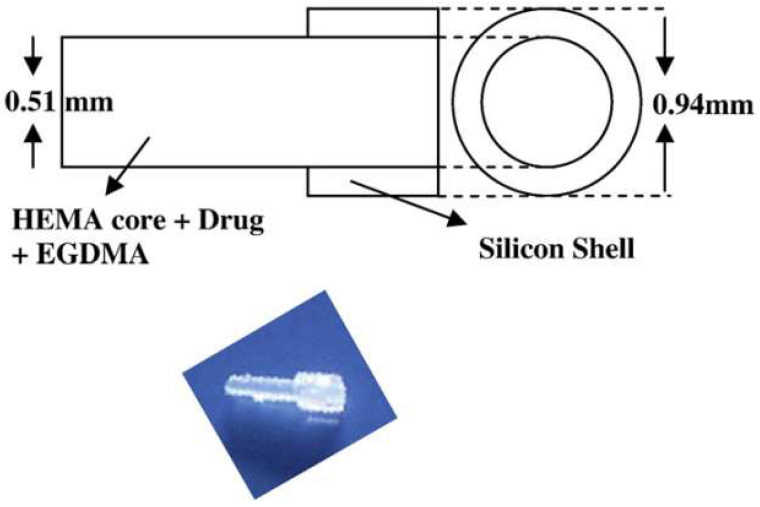
Schematic illustration for the design of a cyclosporine-releasing punctal plug. Reprinted with permission from [94]. Copyright 2011 Elsevier.

Hydrogels have long been used for the fabrication of ocular implants, such as punctal plugs and contact lenses, which also serve as versatile drug delivery vehicles to improve the convenience and effectiveness of DED treatments [19,93]. Gupta et al. developed a cyclosporine-releasing punctal plug with a cylindrical ethyl methacrylate core (Figure 7) [94]. This drug delivery device treats DED through two mechanisms (anti-inflammation and inhibiting tear clearance), thereby providing the patients with a simple and efficient treatment modality. However, the drug in the punctal plug depleted rapidly with low bioavailability. To address this, further efforts have been made by encapsulating drugs in nanocapsules or nanomicelles before embedding them in punctal plugs [95]. Due to the fact that the anatomy of lacrimal ducts varies from person to person, pre-formed punctal plugs are disadvantageous from the perspective of precision medicine. Indeed, complications are frequently reported when the punctal plug does not fit the lacrimal duct of the patient. Xie et al. [96] invented a hyaluronic acid-based in situ punctal plug containing drug-loaded microcapsules. This punctal plug was formed after in vivo injection, thereby fitting lacrimal ducts with different anatomy and circumventing complications associated with conventional punctal plugs. Contact lenses offer another approach for the delivery of DED drugs. To achieve drug encapsulation, the contact lenses are generally dipped in the corresponding drug solution [97,98]. However, this is associated with rapid drug release. Furthermore, some of the encapsulated drugs may make the contact lenses opaque and reduce visual quality. To address these drawbacks, ring-shaped contact lenses have recently been developed, which encapsulate the drug in nanoparticles and deliver it with a ring-shaped boundary region of the contact lenses. For example, prolonged ocular retention and sustained release of hyaluronic acid had been demonstrated with this strategy [99]. It is anticipated that the nanoparticle-encapsulating contact lenses will lead to various innovative therapies for DED.

### 2.9. Others

The anhydrous drug delivery technology (Eyesol^TM^) developed by Novaliq (Baden-Wurttemberg, Germany) is a proprietary technology that eliminates the use of water, oils, surfactants, or preservatives. Hydrophobic drugs can be dissolved easily in the anhydrous solvent to obtain a drug solution that spreads rapidly on the ocular surface to minimize visual disturbances. NOV 03^®^ and CyclASol^®^, two products formulated by this technology, have entered clinical trials. NOV 03^®^ is a single-component eye drop of perfluorohexyloctane. It can penetrate the meibomian glands and dissolve the secretions, thereby stabilizing the lipid layer of tear film [100,101,102]. CyclASol^®^ is developed for aqueous-deficient DED by dissolving cyclosporine in Eyesol^TM^ [103]. Wirta et al. [104] conducted a phase 2 clinical study to evaluate the efficacy, safety, and tolerability of CyclASol^®^ at two different concentrations (0.1% and 0.05% cyclosporine), which revealed that CyclASol^®^ relieved DED symptoms more rapidly than Restasis^®^ and was associated with better outcomes. Based on these promising results, this product is likely to obtain regulatory approval in the near future.

## 3. Conclusions

Various drug delivery systems, particularly suspensions, emulsions, liposomes, nanoparticles, microspheres, hydrogels, and bioadhesive polymers, have been engineered to improve the therapeutic effects of DED drugs. Indeed, promising results are obtained, which have the potential to lead to innovative therapies. Considering the shortcomings of each drug delivery system, the combination of two or more of them deserves further research. Current treatments for DED generally target one aspect of DED pathophysiology. It is fascinating to explore whether superior outcomes can be obtained with drug delivery systems that target multiple aspects of DED pathophysiology simultaneously. There is also a lack of biodegradability and in vivo safety information concerning the above-mentioned drug delivery systems, as most studies are conducted in the short-term setting. A direct comparison between the above-mentioned delivery vehicles represents another issue to be addressed to determine the most suitable one. Finding answers to these questions constitutes the key areas of future research to improve drug delivery for DED.

## Figures and Tables

**Table 1:** Drug delivery systems for DED in the market or under clinical investigation.

Commercial Product	Main Ingredient	Drug Delivery Method	Company	Time to Market
**Restasis^®^**	Cyclosporine A, Polysorbate, Castor Oil, Carbomer, etc.	O/W anionic nanoemulsion	Allergan, Irvine, CA, USA	December 2002
**Cationorm^®^**	Mineral Oil, Glycerin, Tyloxapol, Poloxamer 188, etc.	O/W cationic nanoemulsion	Santen Pharmaceutical, Osaka, Japan	May 2008
**Tears Again^®^**	Soy Lecithin, Sodium Chloride, Vitamin A, Palmitic Acid and Vitamin E, etc.	Liposome spray	Optima Pharmaceutical, Hallbergmoos, Germany	September 2008
**Soothe XP^®^**	Light Mineral Oil (1.0%), Boric Acid, Mineral Oil (4.5%), etc.	O/W anionic nanoemulsion	Bausch & Lomb, Clearwater, FL, USA	May 2010
**Mucosta^®^**	Rebamipide, polyvinyl alcohol, sodium citrate hydrate, sodium chloride.	Suspension	Otsuka Pharmaceutical, Tokyo, Japan	January 2012
**Ikervis^®^**	Cyclosporine A, Medium Chain Triglycerides, Glycerin, Tyloxapol, etc.	O/W cationic nanoemulsion	Santen Pharmaceutical, Osaka, Japan	March 2015
**Cequa^®^**	Cyclosporine A, polyoxyethylene hydrogenated castor oil, etc.	Nanomicelle	Sun Pharmaceutical, Mumbai, India	August 2018
**EYSUVIS^®^**	0.25% Loteprednol Etabonate, etc.	Nanosuspension	Kala Pharmaceuticals, Arlington, MA, USA	October 2020
**Tyrvaya^®^**	varenicline	water-based nasal spray	Oyster Point Pharmaceutical, Princeton, NJ, USA	October 2021
**VisuEvo^®^**	omega-3s, vitamins A and D, and phospholipids, etc.	Liposomes	Visufarma SpA, Rome, Italy	Clinical Trials
**VisuXL^®^**	Coenzyme Q10, Vitamin E TPGS and Sodium Carboxymethylcellulose.	Hydrogel	Visufarma SpA, Rome, Italy	Clinical Trials
**NOV 03^®^**	Perfluorohexyloctane	Anhydrous Drug Delivery System	Novaliq, Baden-Wurttemberg, Germany	Clinical Trials
**CyclASol^®^**	0.1% Cyclosporine A, Semifluorinated alkane	Anhydrous Drug Delivery System	Novaliq, Baden-Wurttemberg, Germany	Clinical Trials

**Table 2:** Bioadhesive polymers for the treatment of DED [82].

Classification	Composition	Product
Natural	Guar Gum	Systane^®^
	Hyaluronic Acid	Hyloforte^®^ Hylocomod^®^ Artelac^®^
Semi-synthetic	Cellulose Derivative	Lacrisert^®^ Systane^®^ Celluvisc^®^
Synthetic	Polyacrylic Acid	Artelac^®^ Vidisic^®^
	Polyvinylpyrrolidone	Protagent^®^ Lacrisic^®^
	Polyvinyl Alcohol	Liquifilm o.k^®^
	Thiolate Compounds	Lacrimera^®^

## Data Availability

All data is contained within the article.
